# Night shift work characteristics are associated with several elevated metabolic risk factors and immune cell counts in a cross-sectional study

**DOI:** 10.1038/s41598-022-06122-w

**Published:** 2022-02-07

**Authors:** Astrid A. Streng, Bette Loef, Martijn E. T. Dollé, Gijsbertus T. J. van der Horst, Inês Chaves, Karin I. Proper, Linda W. M. van Kerkhof

**Affiliations:** 1 Centre for Health Protection, National Institute for Public Health and the Environment (RIVM), P.O. Box 1, 3720 BA Bilthoven, The Netherlands; 2 Department of Molecular Genetics, Erasmus MC Cancer Institute, Erasmus University Medical Centre, Rotterdam, The Netherlands; 3 Centre for Nutrition, Prevention and Health Services, National Institute for Public Health and the Environment (RIVM), Bilthoven, The Netherlands; 4 Department of Public and Occupational Health, Amsterdam Public Health Research Institute, Amsterdam UMC, Amsterdam, The Netherlands

**Keywords:** Biomarkers, Occupational health, Public health

## Abstract

Night shift work is associated with increased health risks. Here we examined the association of metabolic risk factors and immune cell counts, with both night shift work and particular characteristics thereof: frequency, duration and consecutive night shifts. We performed a cross-sectional study using data from 10,201 non-shift workers and 1062 night shift workers of the Lifelines Cohort study. Linear regression analyses, adjusted for demographic, lifestyle and occupational factors, were used to study associations of night shift work characteristics with metabolic risk factors and immune cell counts. Night shift workers had an increased BMI, waist circumference and immune cell counts compared to non-shift workers. This was especially seen in night shift workers who had a higher frequency of night shifts per month (≥ 5: BMI: B = 0.81 kg/m^2^ (95%-CI = 0.43–1.10); waist circumference: B = 1.58 cm (95%-Cl = 0.34–1.71; leukocytes: B = 0.19 × 10^9^ cells/L (95%-CI = 0.04–0.34 × 10^9^)) and worked more consecutive night shifts (> 3: BMI: B = 0.92 kg/m^2^ (95%-CI = 0.41–1.43); waist circumference: B = 1.85 cm (95%-Cl = 0.45–3.24); leukocytes: B = 0.32 × 10^9^ cells/L (95%-CI = 0.09–0.55 × 10^9^)). This association was less pronounced in long-term night shift workers (≥ 20 years). Our findings provide evidence for the association between night shift work characteristics and BMI, waist circumference and leukocytes (including, monocytes, lymphocytes, and basophil granulocytes).

## Introduction

Our current 24-h economy results in frequent occurrence of shift work. In Europe, 19% of the working population works during the night at least once a month^1^. Shift work, and in particular night shift work, is associated with increased health risks, such as cardiovascular and metabolic disorders, increased infection susceptibility, autoimmune diseases, and cancer^2–7^. It is hypothesised that these health risks are a consequence of disturbed circadian rhythms resulting from imposed night shift work^8^. The biological clock—which has a cycle of about 24-h—is the circadian pacemaker of the body and is essential for the timing of physiological processes, and is entrained by light daily^9^. The circadian pacemaker controls among others the sleep–wake cycle, the endocrine and metabolic pathways and the immune system, resulting in oscillation in the composition of immune cells^10–12^.

One challenge of investigating long term health risks of night shift work is that it takes several years to several decades for chronic diseases to develop. It is therefore useful to find risk factors of negative health outcomes on the long term. For metabolic diseases, several predictive markers are well known and have been associated with night shift work^13,14^. For example, a higher BMI increases the chance of developing type 2 diabetes^15–17^, and a higher waist circumference is a strong indicator for abdominal obesity which predicts cardiovascular disease^18–20^. Recently, it was shown that night shift work is also associated with increased immune cell counts^21–25^. Higher levels of, and disturbance of the circadian rhythm in specific types of leukocytes levels in blood, e.g. monocytes or lymphocytes, may be linked with increased inflammation, disease progression and chronic disease risk^26–28^.

Previous studies have indicated that it is important to collect detailed data on the characteristics of the shift work schedule to determine the impact of night shift work on health^29,30^. Characteristics of night shift work (e.g. frequency of night shifts) appear to affect short-term physiological effects and are suggested to also affect long-term health effects^30^. For example, a higher number of night shifts per month has been found to be positively associated with obesity^31–33^, and people working longer than 20 years in night shift work appear to have a lower level of LDL cholesterol and showed a decrease in overweight and obesity^34,35^. Furthermore, recent guidelines also propose to limit the number of consecutive night shifts^30^. However, studies specifically investigating these type of shift work characteristics and the association with risk factors for disease on the long-term are scarce while these studies can be helpful to make more targeted recommendations for the prevention of health problems^30^.

Therefore, the main aim of the current study is to investigate if characteristics of night shift work, i.e. the frequency of night shifts, duration of working in night shifts and number of consecutive night shifts are differentially associated with metabolic risk factors and immune cell counts.

## Methods

### Study population and design

In the current study, we used data from participants of the Lifelines Cohort Study. Lifelines is a multi-disciplinary prospective population-based cohort study examining in a unique three-generation design the health and health-related behaviours of 167,729 persons living in the North of The Netherlands. It employs a broad range of investigative procedures in assessing the biomedical, socio-demographic, behavioural, physical and psychological factors which contribute to the health and disease of the general population, with a special focus on multi-morbidity and complex genetics. The overall design and details on the methodology of the Lifelines cohort study can be found in previous papers^36,37^.

Participants of the Lifelines Cohort study were recruited between 2007 and 2013 and were requested to visit one of the 12 Lifelines research facilities for a basic medical examination between 2014 and 2017. We used available cross-sectional data on the socio-demographic, lifestyle and occupational characteristics, anthropometrics and metabolic and immunological factors of the Lifelines Biobank participants. Data about occupation was collected by questionnaire at the first assessment between 2007 and 2013. For the purpose of the current study, participants of the Lifelines cohort aged ≥ 18 years and with a valid email-address (*n* = 78,190) were approached with an additional questionnaire about their shift work history in December 2017 or January 2018. Questions involved the participants’ shift work history and work schedule during the three months before blood samples were drawn in the second assessment round (2014–2017). The Lifelines study is conducted according to the principles of the Declaration of Helsinki and has been approved by the Medical Ethics Committee of the University Medical Centre Groningen, The Netherlands. Written informed consent was obtained from all participants before entering the study^37^.

### Measures

#### Work schedule

**Figure 1:**
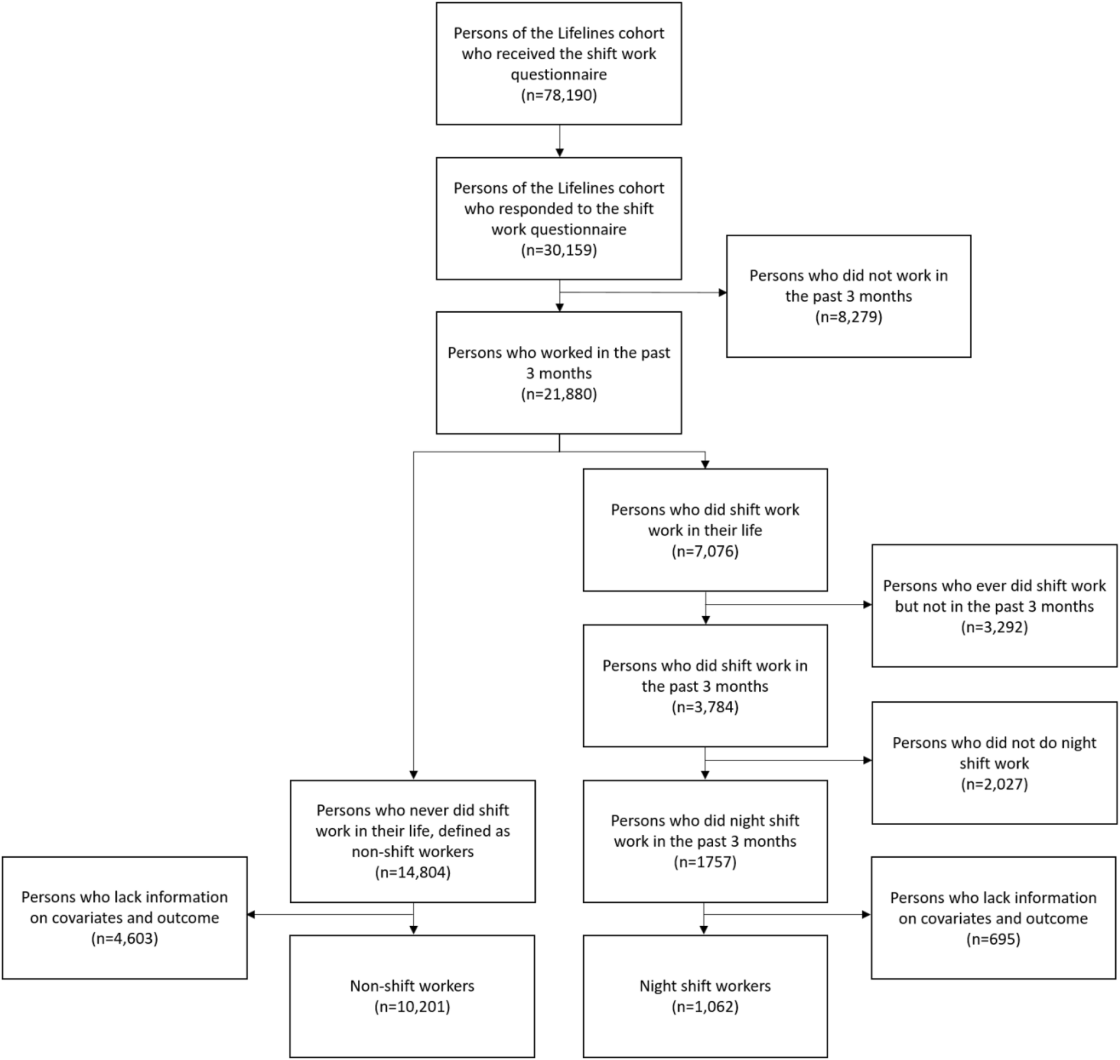
Flowchart Lifelines study population.

In total, 30,159 individuals (38.6%) responded to the shift work questionnaire of whom 21,880 worked at least 12 h per week during the three months before blood samples were taken (Fig. 1). The questionnaire on shift work was designed to cover the major characteristics of shift work: current shift work status, frequency of night shifts (per month), duration of night shift work in years and number of consecutive night shifts (on average during the last 3 months)^30,38^. Night shift workers were defined in case they worked night shifts (either in a rotating or permanent schedule) defined as working at least 3 h between 00.00 and 05.00^38^. Non-shift workers consisted of those who never performed shift work and had regular work times, starting work somewhere after 06.00 and finishing work before 21.00. Average frequency of night shift work per month was categorized into 1–2, 3–4, or ≥ 5 nights shifts/month, duration of night shift work was categorized into < 10, 10–19, or ≥ 20 years of night shift work and the average number of consecutive night shifts was categorized into ≤ 3 consecutive night shifts or > 3 consecutive night shifts^30,39^.

#### Anthropometrics

Data on anthropometrics were obtained from the Lifelines Biobank. Body mass index (BMI) (kg/m^2^) was calculated from measured body weight (kg) and height (m). Waist circumference was measured standing upright with SECA 201 measuring tape which was placed between the lowest rib and the iliac crest around the bare stomach (reading is at 0.5 cm accurate). Blood pressure was measured 10 times during 10 min using a Dinamap (PRO 100V2) automated blood pressure monitor. The average of the final three readings of the blood pressure was registered.

#### Blood parameters and Immunology markers

Participants were asked to fast overnight before blood draw between 8.00 and 10.00 a.m. at one of the Lifelines research facilities. It was not recorded when night shift workers performed their last night shift before blood draw. On the day of collection, total/HDL/LDL cholesterol, triglycerides, glucose and HbA1c were directly processed and measured that same day. Total and HDL cholesterol were measured using an enzymatic colorimetric method, triglycerides with a colorimetric UV method and LDL cholesterol with an enzymatic method (Roche Modular P chemistry analyser). Fasting blood glucose was measured using a hexokinase method. HbA1c concentrations were measured, using a turbidimetric inhibition immunoassay (HPLC, Sysmex corporation). Although participants were requested to fast before the visit, 172 non-shift workers and 28 night shift workers indicated not to have fasted overnight. Blood glucose levels of non- fasting participants were excluded. For the analysis of HbA1c and lipid levels, non-fasted participants were included, as there seems to be no difference between fasting and non-fasting profiles of HbA1c and lipids^40,41^.

Absolute cell counts data of leukocytes was examined and expressed as 10^9^ cells per liter blood. The following cell counts were included: monocytes, lymphocytes, eosinophil granulocytes, basophil granulocytes, neutrophil granulocytes, and thrombocytes. The leukocytes cell counts were measured using Automated Hematology Blood Analyzer (XE2100-system; Sysmex).

The number of individuals included in all analyses is given in Table S1.

#### Covariates

Data on demographics, education, work, family composition, general health, and lifestyle were used as possible confounders, based on the study of Loef and colleagues (2019). To investigate the association with socio-economic factors, the following covariates were included for model 1: age, sex, educational level (low, intermediate or high) and occupation (blue collar, white collar and high skilled or low skilled) based on the ISCO08 categories^42^. To further investigate the association with family composition, general health and lifestyle the following covariates were added to model 2 (fully adjusted): living together with a partner (yes/no), living together with children (yes/no), general perceived health (very good/good/fair vs. mediocre/poor), smoking status (current smoker, former smoker vs. non-smoker), alcohol intake (≤ 7 glasses/week vs. > 7 glasses/week).

#### Statistical analysis

Differences in the main characteristics and variables (metabolic risk factors and immune cell counts) between the night shift workers and non-shift workers were normally distributed and tested with the independent sample t-test for continuous variables and with the chi-square test for categorical variables.

Linear regression analysis was used to study the association between night shift work and each metabolic risk factor and immune cell counts separately, and adjusted in two ways: model 1 (socio-economic covariates) and model 2 (fully adjusted). Subsequently, all analyses were stratified for frequency of night shifts (non-night shift workers, 1–2, 3–4, or ≥ 5 nights shifts/month), duration of night shift work (non-night shift workers, < 10, 10–19, or ≥ 20 years of night shift work), and consecutive night shifts (non-night shift workers, ≤ 3, or > 3 consecutive night shifts). For these analyses, the non-shift workers were used as reference group. Due to the small differences of the association with the addition of covariates between model 1 and 2, it was decided that the stratified analyses were fully adjusted (model 2), for age, sex, educational level, occupation, living together with a partner, living together with children, general perceived health, smoking status, and alcohol intake. Logistic regression was used to evaluate the clinical relevant categories of BMI (< 25 and having overweight ≥ 25 kg/m^2^) and waist circumference (< 80 cm for females, < 94 for males and ≥ 80 for females, ≥ 94 for males) with night shift work. Effect modification was checked for age and sex, by adding interaction terms of the covariates for each outcome measure to the fully adjusted models (interaction was defined as *p* < 0.05). As no statistically significant interaction was observed, results were not stratified for age and sex.

Analyses were carried out using IBM SPSS Statistics V.25.0 (New York: IBM Corp). Statistical significance was defined as *p* < 0.05.

## Results

### Study population

In total 1062 night shift workers (1029 rotating and 33 permanent night shift workers) and 10,201 non-shift workers were included after excluding participants who did not have information on outcomes, exposure, and covariates (Fig. 1). Included participants were a little older, more often males and a bit more often highly educated than non-responders or excluded responders (Table S2). Table 1 shows the main characteristics of night shift workers and non-shift workers. Night shift workers were on average younger (46.7 (SD:8.4) years vs 48.1 (SD:9.1) years) than non-shift workers and underrepresented by females (48.8% vs 53.9%). With respect to occupational status, there were less high skilled white collar workers (52.8% vs 56.2%) and more low-skilled blue collar workers (11.5% vs 5.1%) in the night shift worker group than in the non-shift worker group.

**Table 1:** Characteristics of the study population stratified for night shift workers and non-shift workers.

	Night shift workers (*n* = 1062) % or mean [SD]; median	Non-shift workers (*n* = 10,201) % or mean [SD]; median
Age (in years, mean [SD]; median)	46.7* [8.4]; 48.3	48.1 [9.1]; 49.3
Sex (% female)	48.8*	53.9
**Educational level**		
Lower education (%)	19.1	18.5
Intermediate education (%)	57.3*	36.9
Higher education (%)	23.6*	44.6
Marital status (% living together)	84.1	85.0
Children (% living in the house)	57.9*	52.8
**Smoking status**		
Never smoker (%)	50.7*	56.8
Former smoker (%)	33.1*	30.0
Current smoker (%)	16.3*	13.2
Alcohol intake (% ≤ 7 glasses/week)	70.3	68.2
Perceived health (% fair/good/very good)	93.2	92.9
Occupation**		
White collar high skilled (%)	52.8*	56.2
White collar low skilled (%)	26.7	29.6
Blue collar high skilled (%)	8.5	9.0
Blue collar low skilled (%)	11.5*	5.1

*Statistically significant difference (*p* < 0.05) between shift workers and non-shift workers tested with independent samples t-test or chi-square test.

**In the category “Occupation”, 19 individuals (night shift group *n* = 5; non-shift workers group *n* = 14) are classified as “Armed forces occupations”.

### Metabolic risk factors in night shift workers and non-shift workers

To assess possible differences in metabolic risk factors between night shift and non-shift workers, BMI (continuous and being overweight (BMI ≥ 25 kg/m^2^), waist circumference (continuous and high waist circumference (≥ 80 cm for females, ≥ 94 cm for males), cholesterol (total/HDL/LDL), triglycerides, glucose levels, HbA1c, diastolic and systolic blood pressure were compared between night shift and non-shift workers (Table 2, Fig. S1). The fully adjusted values for BMI (B = 0.57 kg/m^2^ (95%-CI = 0.32–0.82)) and waist circumference (B = 1.03 cm (95%-Cl = 0.34–1.71) were higher in night shift workers than in non-shift workers (Table 2, Fig. S2). In logistic regression analysis, night shift workers were more often overweight (BMI ≥ 25 kg/m2; OR 1.32, 95%-CI = 1.15–1.51) (Table S1). The higher BMI and waist circumference were especially seen in night shift workers with a frequency of ≥ 5 night shifts per month (BMI: B = 0.81 kg/m^2^ (95%-CI = 0.43–1.10), waist circumference: B = 1.58 m (95%-Cl = 0.67–2.49)), as shown in Table 3. The higher level of BMI among night shift workers was observed irrespective of the total years of night shift work (duration), or average number of consecutive night shifts (Table 3). However, the effect size of the increase in BMI and waist circumference was somewhat lower in the night shift group who worked ≥ 20 years in night shifts and higher in the night shift workers who worked on average > 3 consecutive night shifts, as shown in Table 3.

**Table 2:** Differences in metabolic risk factors between night shift workers and non-shift workers.

	Night shift workers^1^ (*n* = 1062) Mean (SD); median	Non-shift workers^1^ (*n* = 10,201) Mean (SD); median	Model 1 Night shift vs. non-shift workers^1,a^ *B (95%-CI)*	Model 2 (fully adjusted) Night shift vs. non-shift workers^1,b^ *B (95%-CI)*
BMI (in kg/m^2^)	26.5** (4.3); 26.0	25.8 (4.0): 25.2	0.58** (0.33–**0.83)**	0.57** (0.32–**0.82)**
Waist circumference (in cm)	91.5** (12.6); 91.0	89.7 (11.9); 89.0	1.08** (0.38–**1.77)**	1.03** (0.34–**1.71)**
Total cholesterol (in mmol/L)	5.05* (0.93); 5.00	5.11 (0.95); 5.10	− 0.04 (− 0.10–0.02)	− 0.04 (− 0.10–0.02)
HDL cholesterol (in mmol/L)	1.46** (0.41); 1.40	1.52 (0.42); 1.50	− 0.02 (− 0.05–0.003)	− 0.02 (− 0.04–0.007)
LDL cholesterol (in mmol/L)	3.31 (0.87); 3.30	3.34 (0.89); 3.30	− 0.03 (− 0.08–0.03)	− 0.03 (− 0.08–0.03)
Triglycerides (in mmol/L)	1.27**(0.79); 1.07	1.20 (0.82); 0.99	0.04 (− 0.02–0.09)	0.03 (− 0.02–0.08)
Glucose (in mmol/L)	5.06 (0.85); 5.00	5.03 (0.75); 4.90	0.01 (− 0.03–0.06)	0.01 (− 0.03–0.06)
HbA1c (in mmol/L)	36.1 (4.55); 36.0	36.05 (4.43); 36.0	0.18 (− 0.09–0.45)	0.15 (− 0.12–0.42)
Diastolic blood Pressure (in mm Hg)	74.8 (9.45); 74.0	74.95 (9.70); 74.00	− 0.39 (− 0.97–0.20)	− 0.36 (− 0.95–0.22)
Systolic blood Pressure (in mm Hg)	127.5 (14.65); 126.0	127.85 (15.26); 126.00	− 0.61 (− 1.53–0.31)	− 0.55 (− 1.46–0.37)

^1^The number of non-shift workers: 10,201 (range between 9990 and 10,199) and night shift workers: 1062 (range between 1029 and 1062). The exact number of individuals included in the analysis is given in Table S1.

^a^Model 1: Adjusted for age, sex, educational level, occupation.

^b^Model 2: Adjusted for age, sex, educational level, occupation, living together with partner, living together with children, general perceived health, smoking, and alcohol intake.

Bold values represent statistically significant difference (* = *p* < 0.05, ** = *p* < 0.01) between night shift workers and non-shift worker with independent samples t- test or linear regression^a/b^.

*B* regression coefficient, *CI* confidence interval, *HDL* high-density lipoprotein, *LDL* low-density lipoprotein.

**Table 3:** Regression coefficients of the differences in metabolic risk factors by average frequency of night shifts, duration of night shift work and average number of consecutive night shifts, compared to non-shift workers***.

	1–2 night shifts/month^1,***^ (*n* = 189) *B (95%-CI)*	3–4 night shifts/month^1,***^ (*n* = 283) *B (95%-CI)*	≥ 5 night shifts/month^1,***^ (*n* = 590) *B (95%-CI)*	< 10 years^1,***^ (*n* = 310) *B (95%-CI)*	10–19 years^1,***^ (*n* = 269)*B (95%-CI)*	≥ 20 years^1,***^ (*n* = 483) *B (95%-CI)*	≤ 3 consecutive night shifts^1,***^ (*n* = 755) *B (95%-CI)*	> 3 consecutive night shifts^1,***^ (*n* = 235) *B (95%-CI)*
BMI (in kg/m^2^)	0.40 (− 0.17–0.96)	0.34 (− 0.18–0.75)	0.81** (0.43–**1.10)**	0.73** (0.28–**1.18)**	0.66** (0.18–**1.14)**	0.41* (0.05–**0.77)**	0.53* (0.23–**0.82)**	0.92** (0.41–**1.43)**
Waist circumference (in cm)	0.95 (− 0.60–2.50)	− 0.04 (− 1.31–1.24)	1.58** (0.67–**2.49)**	1.19 (− 0.04–2.41)	1.22 (− 0.09–2.52)	0.82 (− 1.17–1.81)	0.85* (0.05–**1.66)**	1.85* (0.45–**3.24)**
Total cholesterol (in mmol/L)	− 0.14* (− **0.27 to** − **0.01)**	− 0.02 (− 0.12–0.09)	− 0.01 (− 0.09–0.06)	− 0.06 (− 0.16–0.04)	− 0.009 (− 0.12–0.10)	− 0.04 (− 0.12–0.04)	− 0.04 (− 0.11– 0.03)	0.04 (− 0.08–0.16)
HDL cholesterol (in mmol/L)	− 0.02 (− 0.07–0.04)	0.01 (− 0.03–0.06)	− 0.03* (− **0.06**–**0.000)**	− 0.01 (− 0.06–0.03)	− 0.03 (− 0.08–0.02)	− 0.01 (− 0.05–0.02)	− 0.01 (− 0.04–0.02)	− 0.04 (− 0.09–0.01)
LDL cholesterol (in mmol/L)	− 0.12* (− **0.25 to** − **0.001)**	− 0.02 (− 0.12–0.08)	0.002 (− 0.07–0.07)	− 0.05 (− 0.15–0.04)	0.008 (− 0.10–0.11)	− 0.03 (− 0.11–0.05)	− 0.03 (− 0.10–0.03)	0.06 (− 0.05–0.17)
Triglycerides (in mmol/L)	− 0.008 (− 0.12–0.11)	0.03 (− 0.06–0.13)	0.04 (− 0.02–0.11)	0.01 (− 0.08–0.10)	0.06 (− 0.04–0.15)	0.03 (− 0.04–0.10)	0.03 (− 0.03–0.09)	0.07 (− 0.04–0.17)
Glucose (in mmol/L)	− 0.002 (− 0.11–0.11)	0.005 (− 0.08–0.09)	0.03 (− 0.04–0.09)	0.11* (0.02–**0.19)**	− 0.08 (− 0.17–0.01)	0.007 (− 0.06–0.08)	0.02 (− 0.04–0.07)	0.02 (− 0.08–0.12)
HbA1c (in mmol/L)	− 0.14 (− 0.75–0.46)	0.32 (− 0.18–0.82)	0.17 (− 0.19–0.53)	0.87** (0.39–**1.35)**	− 0.25 (− 0.76–0.26)	− 0.08 (− 0.47–0.31)	0.10 (− 0.22–0.41)	0.37 (− 0.18–0.91)
Diastolic Blood Pressure (in mm Hg)	− 0.67 (− 1.98 − 0.64)	− 0.80 (− 1.88–0.28)	− 0.05 (− 0.82–0.72)	− 0.72 (− 1.76–0.32)	− 0.49 (− 1.60–0.62)	− 0.07 (− 0.90–0.77)	− 0.33 (− 1.01–0.35)	0.14 (− 1.05–1.32)
Systolic Blood Pressure (in mm Hg)	− 0.10 (− 2.16–1.97)	− 1.28 (− 2.98–0.41)	− 0.33 (− 1.54–0.88)	1.00 (− 0.63–2.643)	− 0.64 (− 2.38–1.10)	− 1.47* (− **2.79 to** − **0.16)**	− 0.42 (− 1.49–0.65)	− 0.29 (− 2.15–1.57)

Reference group: non-shift workers.

^1^The number of individuals included in the analysis may differ per variable, the exact number of individuals is given in Table S1.

***Fully adjusted for age, sex, educational level, occupation, living together with partner, living together with children, general perceived health, smoking, and alcohol intake.

Bold values represent statistically significant difference (* = *p* < 0.05, ** = *p* < 0.01) between night shift workers and non-shift worker with linear regression***.

*B* regression coefficient, *CI* confidence interval, *HDL* high-density lipoprotein, *LDL* low-density lipoprotein.

For the remaining metabolic risk factors, i.e. the lipid levels, glucose, and blood pressure measures, there were no overall differences between night shift and non-shift workers before and after adjusting for covariates (Table 2). Stratified analyses for the different characteristics (frequency, duration, consecutive night shifts) of night shift work did not show clear exposure–response patterns for any of these metabolic risk factors. We did observe a few significant differences, for example, the group that worked < 10 years in night shifts. They had a higher fasting glucose level (B = 0.11 mmol/L (95%-CI = 0.02–0.19)) and a higher value of HbA1c (B = 0.87 mmol/L (95%-CI = 0.39–1.35), compared to non-shift workers (Table 3).

### Immune cell counts in night shift workers and non-shift workers

To examine the immune cell counts in night shift workers and non-shift workers, the absolute number (10^9^ cells/L) of leukocytes, monocytes, lymphocytes, granulocytes (eosinophil, basophil and neutrophil) and thrombocytes were investigated (Table 4, Fig. S2). Compared to non-shift workers, the night shift workers showed a higher cell count of leukocytes (B = 0.16 × 10^9^ cells/L (95%-CI = 0.05–0.27)), monocytes (B = 0.02 × 10^9^ cells/L (95%-CI = 0.01–0.03)), lymphocytes (B = 0.12 × 10^9^ cells/L (95%-CI = 0.08–0.15)) and basophil granulocytes (B = 0.001 × 10^9^ cells/L (95%-CI = 0.000–0.003)) in the fully adjusted model (Table 4, Fig. S2).

**Table 4:** Differences in immune cell counts between nightshift workers and non-shift workers.

10^9^ cells/L	Night shift workers^1^ (*n* = 1062) Mean (SD); median	Non-shift workers^1^ (*n* = 10,201) Mean (SD); median	Model 1 Night shift vs. non-shift workers^1,a^ *B (95%-CI)*	Model 2 (fully adjusted) Night shift vs. non-shift workers^1,b^ *B (95%-CI)*
Leukocytes	6.23** (1.72); 6.00	**6.01 (1.82); 5.80**	0.18** (0.06–**0.29)**	0.16** (0.05–**0.27)**
Monocytes	0.52**(0.15); 0.50	**0.49 (0.14); 0.47**	0.02** (0.01–**0.03)**	0.02** (0.01–**0.03)**
Lymphocytes	2.10** (0.70); 2.00	**1.97 (0.59); 1.89**	0.12** (0.08–**0.16)**	0.12** (0.08–**0.15)**
Eosinophil granulocytes	0.20 (0.14); 0.17	0.19 (0.14); 0.16	0.009 (0.000–0.02)	0.008 (-0.001–0.02)
Basophil granulocytes	0.049* (0.02); 0.05	**0.047 (0.02); 0.04**	0.002* (0.000–**0.003)**	0.001* (0.000–**0.003)**
Neutrophil granulocytes	3.34 (1.23); 3.13	3.27 (1.14); 3.08	0.03 (-0.04–0.11)	0.02 (-0.06–0.09)
Thrombocytes	261.6 (61.29) ; 257.0	258.7 (59.44); 253.0	2.79 (-0.95–6.52)	2.74 (-1.00–6.47)

^1^The number of non-shift workers: 10,201 (range between 10,096 and 10,195) and night shift workers: 1062 (range between 1054 and 1062). The exact number of individuals included in the analysis is given in Table S1.

^a^Model 1: Adjusted for age, sex, educational level, occupation.

^b^Model 2: Adjusted for age, sex, educational level, occupation, living together with partner, living together with children, general perceived health, smoking, and alcohol intake.

Bold values represent statistically significant difference (* = *p* < 0.05, ** = *p* < 0.01) between night shift workers and non-shift worker with independent samples t- test or linear regression^a/b^.

*B* regression coefficient, *CI* confidence interval.

The higher level of leukocytes appeared to be mainly due to a higher level of monocytes and lymphocytes among night shift workers. This higher level of monocytes and lymphocytes was observed irrespective of frequency of night shifts, duration of night shift work, or average number of consecutive night shifts per month (Table 5). In general, the effect sizes of these increased cell counts were more pronounced in the group of night shift workers who worked ≥ 5 shifts per month and the group of night shift workers who worked > 3 consecutive night shifts (Table 5). These groups for example had especially a higher cell count of leukocytes (≥ 5 night shifts per month: B = 0.19 × 10^9^ cells/L (95%-CI = 0.04–0.34 × 10^9^), > 3 consecutive night shifts: B = 0.32 × 10^9^ cells/L (95%-CI = 0.09–0.55 × 10^9^), than non-shift workers. With regard to duration of shift work, night shift workers who in total have worked < 20 years in night shifts generally showed increased counts of leukocytes, monocytes and lymphocytes, whereas this association was absent or smaller in size in night shift workers who have worked in night shifts for ≥ 20 years, compared to the non-shift workers (Table 5). For example, the night shift workers who worked less than 20 years in night shifts had a higher cell count of leukocytes than non-shift workers, whereas those working ≥ 20 years in night shift work did not (< 10 years: B = 0.25 × 10^9^ cells/L (95%-CI = 0.05–0.45 × 10^9^), 10–19 years: B = 0.22 × 10^9^ cells/L (95%-CI = 0.004–0.43 × 10^9^), ≥ 20 years: B = 0.07 × 10^9^ cells/L (95%-CI =  − 0.10–0.23 × 10^9^)).

**Table 5:** Regression coefficients of the differences in immune cell counts by average frequency of night shifts, duration of night shift work and average number of consecutive night shifts, compared to non-shift workers***.

10^9^ cells/L	1–2 night shifts/month^1,***^ (*n* = 189) *B (95%-CI)*	3–4 night shifts/month^1,***^ (*n* = 283) *B (95%-CI)*	≥ 5 night shifts/month^1,***^ (*n* = 590) *B (95%-CI)*	< 10 years^1,***^ (*n* = 310) *B (95%-CI)*	10–19 years^1,***^ (*n* = 269) *B (95%-CI)*	≥ 20 years^1,***^ (*n* = 483) *B (95%-CI)*	≤ 3 consecutive night shifts^1,***^ (*n* = 755) *B (95%-CI)*	> 3 consecutive night shifts^1,***^ (*n* = 235) *B (95%-CI)*
Leukocytes	0.17 (− 0.08–0.43)	0.08 (− 0.13–0.29)	0.19* (0.04–**0.34)**	0.25* (0.05–**0.45)**	0.22* (0.004–**0.43)**	0.07 (− 0.10–0.23)	0.12 (− 0.01–0.25)	0.32** (0.09–**0.55)**
Monocytes	0.02 (− 0.001–0.04)	0.02* (0.003–**0.04)**	0.02** (0.009–**0.03)**	0.03** (0.02–**0.05)**	0.02 (− 0.001–0.03)	0.01* (0.001–**0.03)**	0.02** (0.005–**0.03)**	0.04** (0.02–**0.05)**
Lymphocytes	0.10* (0.02–**0.18)**	0.09* (0.02–**0.16)**	0.14** (0.09–**0.19)**	0.15** (0.08–**0.21)**	0.16** (0.09–**0.23)**	0.08** (0.02–**0.13)**	0.11** (0.06–**0.15)**	0.18** (0.11–**0.26)**
Eosinophil granulocytes	0.03** (0.007–**0.05)**	− 0.005 (− 0.02–0.01)	0.008 (− 0.004–0.02)	0.01 (− 0.002–0.03)	0.004 (− 0.01–0.02)	0.006 (− 0.006–0.02)	0.005 (− 0.005–0.02)	0.02 (− 0.002–0.03)
Basophil granulocytes	0.003 (0.000–0.006)	− 0.001 (− 0.003–0.002)	0.002* (0.000–**0.004)**	0.002 (− 0.001–0.004)	0.001 (− 0.001–0.004)	0.001 (− 0.001–0.003)	0.001 (− 0.001–0.002)	0.004** (0.001–**0.007)**
Neutrophil granulocytes	0.04 (− 0.12–0.20)	− 0.03 (− 0.16–0.11)	0.03 (− 0.07–0.13)	0.05 (− 0.08–0.18)	0.04 (− 0.09–0.18)	− 0.02 (− 0.12–0.09)	0.002 (− 0.08–0.09)	0.08 (− 0.07–0.23)
Thrombocytes	9.33* (0.95–**17.71)**	− 2.88(− 9.78–4.03)	3.33 (− 1.60–8.26)	6.71* (0.07–**13.36)**	3.79 (− 3.29–10.87)	− 0.36 (− 5.71–4.99)	0.95 (− 3.39–5.30)	7.61* (0.06–**15.16)**

Reference group: non-shift workers.

^1^The number of individuals included in the analysis may differ per variable, the exact number of individuals is given in Table S1.

***Fully adjusted for age, sex, educational level, occupation, living together with partner, living together with children, general perceived health, smoking, and alcohol intake.

Bold values represent statistically significant difference (* = *p* < 0.05, ** = *p* < 0.01) between night shift workers and non-shift worker with linear regression***.

*B* regression coefficient, *CI* confidence interval.

## Discussion

In this study, it was investigated whether the characteristics of night shift work, i.e. the frequency of night shifts, duration of working in night shifts and number of consecutive night shifts, are associated with metabolic risk factors and immune cell counts. Night shift workers had a higher BMI, were more often overweight (BMI ≥ 25 kg/m2), had a higher waist circumference and higher levels of several immune cell types (leukocytes, monocytes, lymphocytes and basophil granulocytes counts) than non-shift workers. Several of these differences were especially seen in night shift workers who worked ≥ 5 nights per month, < 20 years of night shift work in total, or on average > 3 consecutive night shifts.

The observed higher BMI and waist circumference among shift-workers in our study is in line with several prior studies^13,14,31,43,44^. It indicates that night shift workers may be more prone to develop several diseases, such as type 2 diabetes, cardiovascular disease, hypertension, and cancer^17,45^. Previously, several mechanisms for the relationship between night shift work and increased BMI or waist circumference have been proposed, such as reduced physical activity^46^, higher caloric intake^47^, and shorter sleep duration or other mechanisms related to circadian disturbances^44,48–50^. Future research, e.g. in a longitudinal setting, is needed to examine whether these proposed mechanisms underlie the observed higher BMI and waist circumference among night shift workers.

In this study, no association was found between night shift work and lipid levels, blood glucose and HbA1c, blood pressure and high waist circumference (≥ 80 cm for females, ≥ 94 cm for males). A previous meta-analysis of Proper and colleagues (2016) concluded that there is insufficient evidence for an association between shift work and a change in glucose, HBA1c, lipid levels, and blood pressure. Based on these and our findings it is, at the moment, difficult to draw a firm conclusion on the association between night shift work and these specific metabolic factors.

In our study, counts of leukocytes were higher among night shift workers than non-shift workers, with specifically a higher number of monocytes, lymphocytes and basophil granulocytes, which is in line with several other studies^21,22,24,25,51^. There is growing evidence that having increased levels of (differential) leukocytes is a risk factor for type 2 diabetes^52–55^, cancer^56–58^, and cardiovascular diseases^26,59–61^. Shift workers are thought to suffer disproportionally from all of the aforementioned diseases^62–64^. For example, the observed elevation in monocytes that are observed in night shift workers, are proposed to play a mechanistic role in these negative health effects^25,65^. The infiltration of monocytes can modulate atherosclerosis progression, but also causes progression of tumour metastasis^65^. Leukocytes are known to play diverse roles in the body during health and disease, for example, cytokines produced by leukocytes can have multiple effects. Proinflammatory cytokines can promote tumour growth, while anti-inflammatory cytokines interfere with tumour growth^57^. This may make increased BMI, waist circumference and changes in leukocytes (including, monocytes, lymphocytes, and basophil granulocytes) a possible underlying mechanism of adverse health effects in night shift workers, which is a relevant topic for further studies.

### Frequency of night shifts per month

In our study, it was notable that associations of night shift work with metabolic risk factors and immune cell counts were in particular present or higher in the group of night shift workers with the highest frequency of night shifts per month (i.e. ≥ 5 night shifts). For example, this was observed for BMI, waist circumference and lymphocytes. For the metabolic risk factors, this is in line with several previous studies^31–33^. The findings in our study and previous studies^31–33^, indicate that in particular frequent shift workers are at increased risk of having a higher BMI and higher waist circumference, which may be associated with a higher risk of developing diseases. This suggests that limiting the number of night shifts per month could be an opportunity to decrease health risks in night shift workers.

Night shift workers who have on average a higher frequency of night shifts showed a higher level of lymphocytes. This group is more likely to recently have done night shift work at the moment of blood collection. In the study by Loef and colleagues (2019), it was suggested that the observed increased lymphocyte cell count in night shift workers was due to an acute effect of night shift work, since it was only observed in night shift workers who performed a night shift in the past 3 days, in contrast to monocytes which were elevated in night shift workers irrespective of working night shifts in the past 3 days. Therefore, it is possible that in our study the increase in lymphocytes observed particularly in frequent shift workers (≥ 5 night shifts) is (partially) related to an acute effect of night shifts.

### Duration of night shifts in years

For BMI, waist circumference, and several immune cell types, we observed no or a weaker association in the group with the longest duration of night shift work, i.e. night shift workers who worked in night shifts for ≥ 20 years. One likely explanation for this finding could be the ‘healthy worker effect’^66,67^. This refers to the effect that individuals who are better able to tolerate night shift work are more likely to remain in night shift work. The ‘healthy worker effect’ is seen in several studies. For example, Loef, et al.^35^ found that LDL cholesterol is lower in night shift workers working longer than 20 years in night shifts. Also, Pan and colleagues (2011) found a decrease in overweight and obesity in night shift workers working longer than 20 years in night shifts compared to the night shift workers who worked 10–19 years in night shifts^34^. On the other hand, there are studies that show an association between metabolic risk factors and a longer duration of night shift work. For example, in the meta-analysis of Sun and colleagues (2018) on the relationship between night shift work and obesity related measures, some studies indicated increased effects with longer duration in shift work^31,68,69^. Although this effect is dependent on the type of obesity measure (BMI: 25–29.99 kg/m^2^, BMI: ≥ 30 kg/m^2^, abdominal obesity) and how duration in shift work is defined. Similar categories for duration of shift work were used in the study done by Peplonska and colleagues (2015) who found a higher OR of 1.5 in obesity (BMI > 30 kg/m^2^) among night shift workers who worked for ≥ 20 years in night shift work compared to the group of shift workers who worked < 10 years in night shifts. However, Peplonska and colleagues (2015) did not find a significant difference between BMI and duration of night shift work (per 10 years).

We observed a higher glucose and HbA1c in night shifts workers who worked for < 10 years in night shifts, whereas this association was not observed in shift workers working in night shift for 10–19 years or ≥ 20 years. Interestingly, Vetter and colleagues (2018) found an association between shorter durations of night shift work exposure (i.e. < 10 years) with higher type 2 diabetes incidence, whereas this association was not found in long term night shift workers (i.e. > 10 years). For immune cell counts, the smaller effect sizes in groups with longer duration of night shift work of leukocytes and lymphocytes was not observed by Loef and colleagues (2019). The difference in findings for the immune cell counts might be due to the variations of the characteristics of (night shift) work, e.g. sex and/or occupation. In the study of Loef and colleagues (2019), females in the care and health sector were recruited, while in our study individuals were working in different occupational sectors.

As the few studies done so far on duration of shift work and the association with metabolic or immunological factors are inconsistent, additional evidence is necessary to draw conclusions on the association with duration of night shift work on these risk factors.

### Consecutive night shifts

It has been advised to limit the number of consecutive night shifts to a maximum of 3 consecutive nights as it is suggested that this results in a lower risk of accidents and possibly cancer (e.g. breast)^30,39^. The number of consecutive night shifts is thought to have an impact on sleep debt and to have an effect on the extent of desynchronization of the diurnal rhythm, with little room for adaptation^70^. In our study, associations with metabolic risk factors and higher immune cell counts were, in general, more pronounced in night shift workers working > 3 consecutive night shifts. For example, BMI and waist circumference were particularly higher in > 3 consecutive night shifts, as well as the level of leukocytes. To our knowledge, this is the first study demonstrating the association between ≤ 3 consecutive or > 3 consecutive night shifts and elevated metabolic and immune parameters in night shift workers. Together, the higher metabolic risk factors and higher immune cell counts might indicate that a higher number of consecutive night shifts might be associated with higher risk of adverse health effects. This finding could be taken into consideration by employers to optimize night shift work schedules in a way that reduces the health risks. For this, it would be helpful if more studies (including longitudinal studies) examine the number of consecutive night shifts in relation to long term adverse health effects and its risk factors.

### Limitations and strengths

All characteristics and blood samples were collected during the second assessment round of the Lifelines cohort study (2014–2017), while the questionnaire about (shift) work history was taken retrospectively (between December 2018 and March 2019), which could create bias. This recall bias could have especially occurred in the questions about frequency, duration of night shift work and number of consecutive nights shifts, as these might be more difficult for participants to remember than simply doing night work (yes/no). However, a major strength is that this is the first study that examines the association of number of consecutive night shifts with metabolic risk factors and immunological cell counts in night shift workers. The Lifelines population is a representative cohort of the population in the North of the Netherlands and a wide range of professions are taken into account. However, our current study includes a selection of the participants of the Lifelines population. Therefore, selection bias might have occurred. As described in the results section, included participants were a little older, more often males and a bit more often highly educated than non-responders or excluded responders (Table S2). Another limitation of our study is that it is unknown whether the night shift workers had recently worked in a night shift before blood was collected, so we were unable to disentangle acute and more chronic associations. Also, it was not documented whether people were ill or suffering from an infection around the time of blood collection. In future research, these aspects should ideally be taken into account. An additional limitation is that the data about occupation was collected during the first assessment (between 2007 and 2014), which could create incorrect data about occupation as participants could have changed jobs in the meantime. Although it is unlikely that workers change between high or low skilled and blue or white collar work. Also, it is important to note that there is a close relationship between night shift schedule and other occupational and/or lifestyle conditions. Compared to the night shift workers who worked 1–2 night shifts per month, the night shift workers who worked ≥ 5 night shifts per month are more often males (70.0% vs. 31.7%), more often lower educated (24.9% vs. 13.2%), more often working in the technical sector (35.9% vs. 9.0%) and less often working in the care and health sector (28.5% vs. 69.3%). We adjusted our analyses for sex, education, and occupation, but potential residual confounding due to other differences in individual and occupational factors cannot be completely ruled out. The current study has a cross-sectional design, limiting the possibilities for assessing cumulative exposure and causal relationships. In the future, a longitudinal study taking into account the characteristics of night shift work like in our study, could be considered to further enhance our understanding on the relationship with metabolic and immunological risk factors.

## Conclusion

The current study provides further evidence for the association between night shift work and BMI, waist circumference and leukocytes (including, monocytes, lymphocytes, and basophil granulocytes). Our study indicates that risk factors appear to be especially increased in night shift workers who had a higher frequency of night shifts (≥ 5 night shifts per month) and worked more consecutive night shifts (> 3 consecutive night shifts), while these associations were less pronounced in long-term night shift workers (≥ 20 years). Further studies investigating the effect of reduced number of night shifts per months and consecutive night shifts might be beneficial for developing healthier night shift work schedules.

## Supplementary Information

Supplementary Information.

## Data Availability

Data may be obtained from a third party and are not publicly available. Researchers can apply to use the Lifelines data used in this study. More information about how to request Lifelines data and the conditions of use can be found on their website (https://www.lifelines.nl/researcher/how-to-apply).
