# Supplementary material for: Night shift work characteristics are associated with several elevated metabolic risk factors and immune cell counts in a cross-sectional study
Source: Sci Rep. 2022 Feb 7;12:2022. doi: 10.1038/s41598-022-06122-w (PMC8821707; doi:10.1038/s41598-022-06122-w)
Supplement: Supplementary file 1 — Supplementary Information. [file 41598_2022_6122_MOESM1_ESM.docx]

**Supplementary materials to Streng et al.**

**Table S1.** Number of people that were included in the analyses in Table 2-5.

|  | Non-shift workers | Night shift workers | 1–2 night shifts/  month | 3–4 night shifts/  month | ≥5 night shifts/  month | <10 years | 10–19 years | ≥20 years | ≤3 consecutive night shifts | >3 consecutive night shifts |  |
| --- | --- | --- | --- | --- | --- | --- | --- | --- | --- | --- | --- |
| BMI | | 10,197 | 1,062 | 189 | 283 | 590 | 310 | 269 | 483 | 755 | 235 |
| Waist circumference | | 10,196 | 1,062 | 189 | 283 | 590 | 310 | 269 | 483 | 755 | 235 |
| Total/HDL/LDL cholesterol and Triglycerides | | 10,199 | 1,062 | 189 | 283 | 590 | 310 | 269 | 483 | 755 | 235 |
| Glucose | | 9,990 | 1,029 | 181 | 273 | 575 | 303 | 262 | 464 | 726 | 233 |
| HbA1c | | 10,190 | 1,062 | 189 | 283 | 590 | 310 | 269 | 483 | 755 | 235 |
| Diastolic/Systolic Blood Pressure | | 10,195 | 1,062 | 189 | 283 | 590 | 310 | 269 | 483 | 755 | 235 |
| Leukocytes | | 10,195 | 1,062 | 189 | 283 | 590 | 310 | 269 | 479 | 755 | 235 |
| Monocytes | | 10,098 | 1,054 | 188 | 283 | 583 | 308 | 267 | 479 | 750 | 232 |
| Lymphocytes | | 10,096 | 1,054 | 188 | 283 | 583 | 308 | 267 | 479 | 750 | 232 |
| Eosinophil granulocytes | | 10,097 | 1,054 | 188 | 283 | 583 | 308 | 267 | 479 | 750 | 232 |
| Basophil granulocytes | | 10,097 | 1,054 | 188 | 283 | 583 | 308 | 267 | 479 | 750 | 232 |
| Neutrophil granulocytes | | 10,098 | 1,054 | 188 | 283 | 583 | 308 | 267 | 479 | 750 | 232 |
| Thrombocytes | | 10,195 | 1,062 | 189 | 283 | 590 | 310 | 269 | 483 | 755 | 235 |

**Table S2.** Characteristics of the study population stratified for responders and non-responders and excluded responders^1^.

|  | Responders  (n=11,263)  % or mean [SD]; median (n) | Non-responders and excluded responders  (n=36,679)  % or mean [SD]; median (n) |
| --- | --- | --- |
| Age (in years, mean [SD]; median (n)) | 48.0* [9.0]; 49.2 (11,263) | 45.9 [10.1]; 47.0 (n=32,443) |
| Sex (% female (n)) | 53.4* (6,016) | 56.3 (20,648) |
| Educational level | | |
| Lower education (% (n)) | 18.3 (2,066) | 18.9 (5,489) |
| Intermediate education (% (n)) | 38.4* (4,324) | 39.7 (11,518) |
| Higher education (% (n)) | 42.2* (4,753) | 40.0 (11,621) |

^1^ Only working population is included

*Statistically significant difference (p<0.05) between responders and non-responders and excluded responders tested with independent samples t-test or chi-square test.

**Table S3.** The logistic regression analysis of overweight and high waist circumference on night shift work.

|  | Odds ratio (OR) | 95% Confidence interval |
| --- | --- | --- |
| Overweight (≥25 kg/m^2^) | **1.32**** | **1.15-1.51** |
| High waist circumference (≥80 cm for females, ≥94 cm for males) | 1.06 | 0.92-1.21 |

Bold values represent statistically significant difference (*= p<0.05, **= p<0.01) between night shift workers and non-shift worker with binary logistic regression.

**Figure S1.** Differences in metabolic risk factors between night shift workers and non-shift workers.

B, regression coefficient; CI, confidence interval; HDL, high-density lipoprotein; LDL, low-density lipoprotein.

**A**

**B**

**C**

**D**


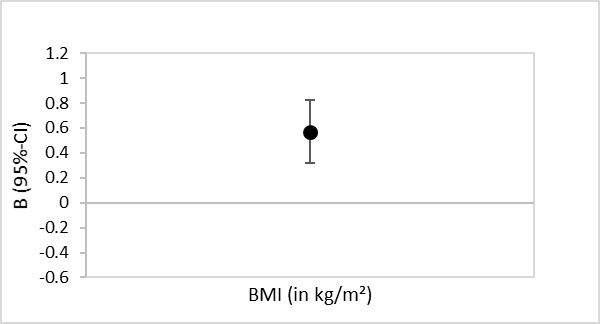

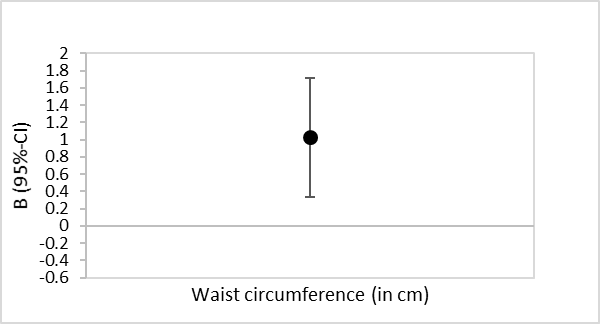

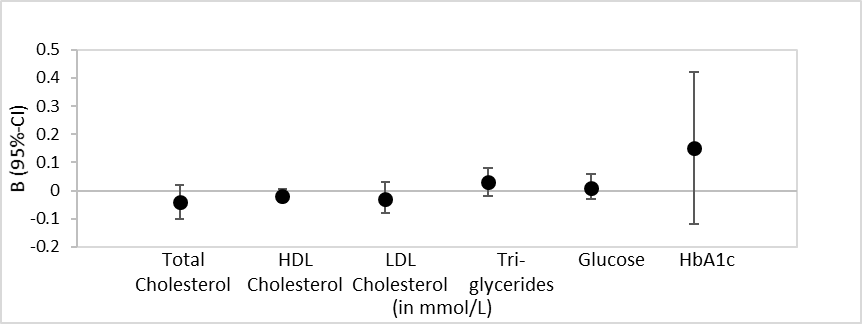

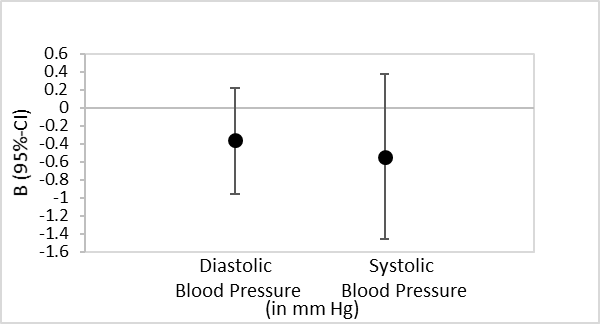


**Figure S2.** Differences in immune cell counts between nightshift workers and non-shift workers.

B, regression coefficient; CI, confidence interval.


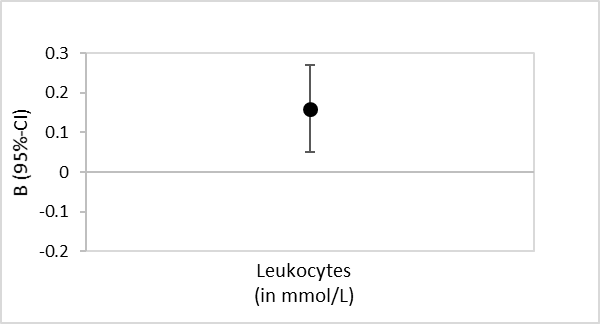

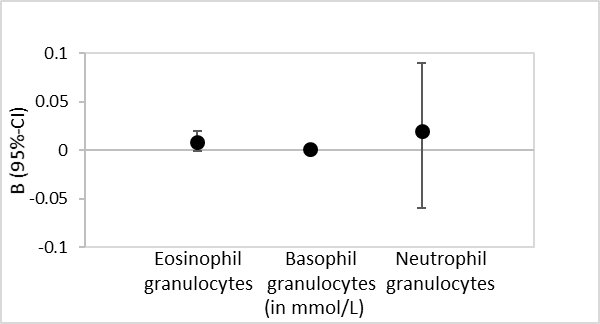

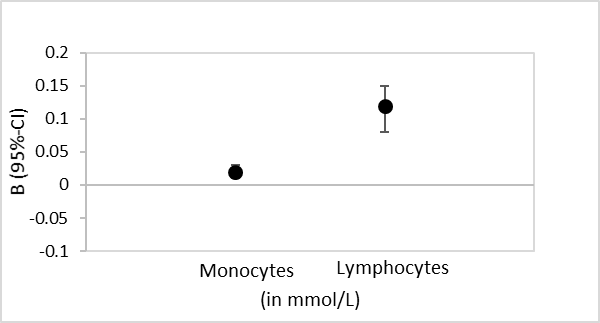

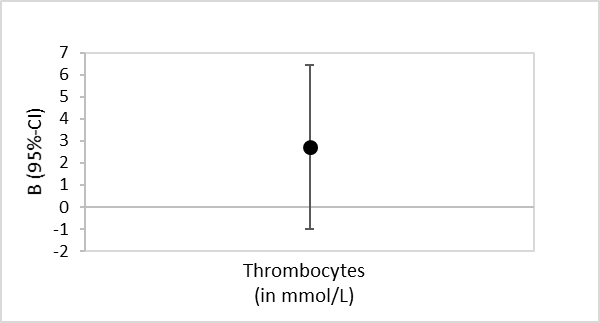


**D**

**C**

**B**

**A**
